# A Decade of Progress: Assessing Three Revascularization Strategies for Iliac Occlusive Disease Through a 580-Case, 10-Year-Experience Literature Comparison

**DOI:** 10.7759/cureus.66826

**Published:** 2024-08-13

**Authors:** Bistra Boneva, Boris Ilchev, Margaret Dimova, Mario Stankev

**Affiliations:** 1 Vascular Surgery, National Hospital of Cardiology, Sofia, BGR; 2 Vascular Surgery, Acıbadem City Clinic Tokuda Hospital, Sofia, BGR; 3 Vascular Surgery, University Hospital Lozenetz, Sofia, BGR

**Keywords:** aorto-iliac occlusive disease, open surgery, endovascular surgery, hybrid surgery, iliac occlusive disease

## Abstract

Introduction:* *Peripheral arterial disease (PAD) is a critical concern, particularly in the context of an aging population and escalating risk factors such as diabetes, hypertension, and smoking. PAD leads to significant morbidity and disability, imposing considerable healthcare and economic burdens. A detailed understanding of the functional outcomes of revascularization is essential as it influences the choice of therapeutic strategies. This is crucial for the patient-doctor dialogue, enabling informed decisions based on the benefits, risks, and costs associated with each option. This study specifically examines the effectiveness of various revascularization methods for iliac occlusive disease by analyzing factors such as procedural success rates, complication frequencies, long-term patency, and patient quality of life. By evaluating these characteristics, the study aims to guide surgeons in selecting the most appropriate treatment approach in modern vascular surgery.

Methods: A 10-year single-center retrospective analysis was conducted, examining 521 patients (580 interventions) from January 2009 to December 2018. Treatments included endovascular recanalization and stenting (endovascular treatment, EVT) (31.4%), hybrid surgical treatment (HST) (31.6%), and open surgical treatment (OST) (37.07%). The examined characteristics were primary patency, primary assisted patency, secondary patency, complications, and the degree of limb salvage.

Results: The study assessed variables such as age, gender, diabetes, hypertension, dyslipidemia, smoking status, chronic kidney disease, and anesthesiological risk (American Society of Anesthesiologists (ASA) grade). Patency rates across the three methods were 92.4%, with thrombosis observed in 7.6% of cases. Assisted primary reconstructions, identified in the analysis, were few in number. Across the three revascularization strategies, a total of 41 interventions were undertaken to preserve the patency of the index reconstruction. In cases of chronic limb-threatening ischemia (CLTI), the probability of losing patency is higher and occurs earlier. OST showed the longest patency duration (471.7±71.5 days), and EVT demonstrated consistent primary patency. Complications were the highest in OST, including five perioperative mortalities. Survival analysis revealed significant differences in patency between treatment methods, with EVT and HST showing better outcomes compared to OST, particularly in patients with CLTI.

Conclusion: By far, this is one of the largest studies done comparing all three revascularization strategies. Endovascular, surgical, and hybrid interventions should be considered complementary elements in the vascular surgeon's toolkit. However, in the presented study, endovascular and hybrid treatment appeared to produce better outcomes compared to open surgical treatment, especially in patients with CLTI. Keeping this in mind the surgeon should be able to provide a more optimal and personalized treatment for patients with chronic lower limb ischemia.

## Introduction

Cardiovascular diseases (CVD) represent a significant public health issue, ranking as the leading cause of mortality worldwide. The multifocal nature of the underlying atherosclerotic process makes these diseases particularly complex and multifaceted, both in terms of understanding and treatment. Such conditions involve the coronary circulation, cerebrovascular circulation, and limb blood supply as part of peripheral arterial disease (PAD). PAD is a major cause of loss of the ability for pain-free movement, posing a significant challenge for vascular specialists. Besides affecting the lower limbs it is well known that PAD is associated with higher systemic morbidity and mortality. The term PAD encompasses a constellation of diseases, leading to atherosclerosis-induced stenoses or occlusions of major vessels, causing insufficient blood supply to the tissues of the lower limbs. The symptomatology of the disease primarily manifests as muscle pain in the lower limb, dependent on the degree of underlying ischemia. Pain ranges from claudication to rest pain in cases of chronic limb-threatening ischemia (CLTI) [1]. Vascular surgery has undergone significant changes and developments over the years, introducing new treatment techniques [2]. Despite this progress, there are challenges in systematizing and optimizing treatment algorithms for various vascular pathologies.

In the modern medical world, the influence of industry and the evolution of technologies are factors that cannot be halted. They inevitably transform treatment strategies. Technological innovations allow surgeons to perform precise interventions using minimally invasive methods that reduce surgical trauma, shorten patients' recovery time, and consequently reduce hospital stay, but they might not be the best long-term option. However, vascular surgeons face the challenge of mastering all these diverse surgical techniques and choosing the right treatment method for the specific medical case.

We believe that in today's medical community, it is unthinkable to bypass the rules of good medical practice or ignore the guidelines [3]. These are constantly updated based on randomized and controlled studies, aiming to practice evidence-based medicine. This retrospective study aims to contribute to a better understanding of the multifactorial disease process and the choice of an alternative surgical approach. The treatment of PAD has always been guided by the patient's overall condition and the stage of the disease. The choice of treatment strategy varies depending on whether the patient is a claudicant, a patient with CLTI, or a patient with the onset of tissue loss from the distal part of the limb.

This study aims to present a comparative analysis of revascularization treatment methods for iliac occlusive disease, examining the effectiveness and advantages of different approaches in contemporary vascular surgery.

## Materials and methods

A single-center retrospective analysis was conducted at the Vascular Surgery Clinic of the National Cardiology Hospital, Sofia, over a 10-year period, which spanned from January 2009 to December 2018. A total of 521 patients who underwent 580 surgical interventions were selected. The number of interventions exceeds the number of patients due to the long study period and the multifocal nature and progression tendency of the atherosclerotic disease process. Many patients received different treatments for each limb at different times, leading to their reconstructions being treated as separate events in the analysis. These are categorized according to the treatment method applied: endovascular recanalization and stenting of the iliac artery (endovascular treatment (EVT)), 182 (31.4%); hybrid surgical treatment (HST), 183 (31.6%); and classic open surgical treatment (OST), 215 (37.1%). The average age of the operated patients was 64.4 years +/- 8.8, with the highest frequency of patients between 60 and 80 years.

The stage of PAD was assessed using the Fontaine clinical classification [3], which is embedded in everyday practice in Bulgaria. All studied subjects were candidates for revascularization in clinical stage 2B, 3, or 4. All patients in the study population had angiographically or CT-proven lesions falling into the Trans-Atlantic Inter-Society Consensus (TASC) II C and D categories for iliac lesions.

The follow-up protocol for the intervened patients included assessment of palpable femoral pulse and recording of clinical improvement in symptomatology. Follow-up visits were scheduled at 1, 3, 6, 12 months, and annually thereafter, in the absence of disease stage worsening or acute loss of patency.

Exclusion criteria

Patients with TASC class D lesions affecting the infrarenal aorta were excluded from the study (Leriche syndrome, concomitant abdominal aneurysm, severe atherosclerotic involvement extending above the inferior mesenteric artery). Also excluded were patients with short segmental lesions (stenoses and occlusions) under 5 cm, treated only with balloon angioplasty and with a good post-procedural outcome without the need for stent implantation. Patients undergoing emergency surgical interventions were also not included in the sample. Patients with bilateral iliac involvement were operated on in two stages, with each procedure entered and interpreted separately in the study.

Definitions

Technical success: A procedure is considered technically successful if it is completed without complications and has restored the patency of the vessel or the main blood flow to the lower limb, with post-procedural patency present for at least 30 days.

Loss of patency: Loss of patency of the reconstruction is acknowledged in the presence of a loss of the femoral pulse, return of symptoms, ultrasound-proven stenosis or occlusion in the reconstructed arterial segment, or a combination of these findings. Patients suspected of iliac or in-stent restenosis are rehospitalized, angiographically evaluated, and reintervened. This is how the primary, primary-assisted, and secondary patency of reconstructions are assessed.

Technically successful endovascular procedure: An endovascular revascularization that has provided good inflow, ensured outflow at least to the deep femoral artery, and without subsequent open surgical procedures. The need for additional endovascular corrections is not considered a technical failure of the EVT.

Primary patency: It is defined as the patency of the reconstruction without any reinterventions.

Assisted primary patency: It refers to the presence of a subsequent intervention aimed at maintaining the patency of the existing reconstruction.

Secondary patency: It is defined after a reintervention due to occlusion of the existing reconstruction.

Restenosis: It is defined as an ultrasound-measured ratio of peak velocities before and after the stenosis of over 2.5 or angiographically verified stenosis of over 50%.

Clinical/procedural success: It refers to an improvement in the patient's condition by at least one stage of PAD.

Statistics

The data were processed with a specialized Statistical Product and Service Solutions (SPSS, version 20.0; IBM SPSS Statistics for Windows, Armonk, NY). The critical level of significance was α=0.05. The null hypothesis was rejected when the p-value was less than α. Мethods of descriptive statistics where continuous data were expressed as means and standard deviations, while categorical data were presented as absolute values and percentages; chi-square analysis evaluates the association between categorical variables to determine if observed frequencies differ from expected frequencies; Kaplan-Meier analysis estimates survival probabilities over time for a given cohort, accounting for censored data; log-rank test compares survival distributions between two or more groups to assess statistically significant differences; Wilcoxon signed rank tests for the differences between paired or matched samples, focusing on the direction and magnitude of differences, were applied.

Surgical methods for revascularization

Open Surgical Interventions (OST)

OST were performed under general anesthesia using either transperitoneal or retroperitoneal access. Dacron silver prostheses with appropriate diameters (7-8 mm), comparable to the diameter of the patient's target vessels, were used. After open surgical treatment, patients were prescribed mandatory lifelong antiplatelet therapy with either acetylsalicylic acid (100 mg once daily) or clopidogrel (75 mg once daily), applied in cases without a cardiogenic reason or other indication for anticoagulant use.

EVT

Endovascular approaches in the treatment of PAD have rapidly evolved, driven by the minimal invasiveness of the procedure, reduced surgical trauma, fast improvement of new endovascular devices, growing experience among operators, and competitive advancements in medical technology. The choice of EVT as a treatment strategy for iliac occlusive disease (IOD) is strongly influenced by the anatomy and characteristics of the vessel. Challenges arise with heavily calcified or tortuously elongated common iliac arteries (CIA) and when the lesion involves the artery's orifice, potentially necessitating the implantation of a stent in the contralateral iliac artery (kissing technique). The external iliac artery (EIA) may be tortuous, further complicating lesion traversal. All endovascular procedures included in this study were performed by certified vascular surgeons under angiographic control in an operating room equipped with a C-arm. Brachial, ipsi-, or contralateral femoral puncture access was used, depending on the anatomical pattern of the target lesion, under local infiltration anesthesia.

The post-dilatation result was angiographically verified in three projections. In cases of rapid recoil or flow-limiting dissection, the arterial segment was indicated for stenting. Balloon-mounted stents were used for heavily calcified lesions and those near the aortic bifurcation. Self-expanding stents were utilized for lesions at the transition between the common and EIA and for lesions encompassing the EIA. Covered stent grafts were not routinely implanted within the scope of this study, except for one patient where endovascular recanalization of the EIA was complicated by rupture. This patient remained in the study group due to the implantation of an uncovered stent in the proximal part of the iliac segment. After a successful endovascular procedure with stent implantation, dual antiplatelet therapy with acetylsalicylic acid (100 mg) and clopidogrel (75 mg) was initiated for at least three months, after which patients continued lifelong medication with a single antiplatelet agent.

HST

EVT applied to the EIA is associated with low morbidity and mortality but has a higher rate of restenosis and reinterventions compared to OST, which shows excellent long-term patency of the reconstruction but has more complications and mortality. Most recent publications recommend primary EVT even for complex lesions in the aortoiliac segment. However, the results are disappointing in cases of advanced involvement of the EIA and simultaneous involvement of the common femoral artery (CFA). For these patients, remote iliac endarterectomy presents a less invasive hybrid alternative to surgical bypass. In well-selected patients, this technique combines the advantages of open and endovascular surgery.

Surgical technique: A ring stripper of appropriate size is placed retrogradely from the prepared CFA according to the specific anatomy of the patient. The endarterectomy extends to the orifice of the hypogastric artery and, in some cases, to the level of the CIA if the orifice of the internal iliac artery does not create resistance. In some cases, MollRing Cutter (LeMaitre Vascular, Inc., Burlington, MA), which allows a clean cut of the specimen in its proximal part, was used. Subsequently, the specimen is evacuated through the arteriotomy by gently pulling the stripper. In some patients, endarterectomy with the ring stripper was performed over a guidewire previously placed in the aortic lumen, making the procedure safer in clinical cases of adventitia breach requiring rapid stent graft implantation. Following the evacuation of the atherosclerotic plaque, intraoperative angiography was performed. All residual high-grade stenosis in the CIA or its transition with the EIA underwent angioplasty with or without stenting. The size of the stent was determined by the proximal arterial segment to avoid overstretching, with stent sizes ranging from 6 to 9 mm. Self-expanding stents were typically used at the transition between the common and external iliac artery.

## Results

Preoperative Profile of the Studied Cases

The profile of the cases subjected to the three treatment methods is based on descriptive statistics that define the characteristics of the analyzed variables: gender, age, presence of diabetes and its treatment type, presence and degree of arterial hypertension (AH), dyslipidemia, smoking status, chronic kidney disease (CKD), and anesthesiological risk derived (American Society of Anesthesiologists (ASA) grade). Dependencies between some of these indicators and the applied treatment method were established using chi-square analysis. The obtained results are summarized in Table 1.

**Table 1:** Preoperative profile of the studied patients EVT-Endovascular treatment; HST-Hybrid surgical treatment; OST-Open surgical treatment; ASA grade-American Society of Anesthesiologists Grade; PAD stage (Fontaine)-Peripheral Artery Disease Stage by Fontaine Classification

INDICATOR			EVT	HST	OST	TOTAL	STATISTICAL METHOD
FEMALE			49 (47.1%)	32 (30.8%)	23 (22.1%)	104 (100%)	Pearson Chi-Square = 17.669 Asymp. Sig. (2-sided) = 0.000
MALE			133 (27.9%)	151 (31.7%)	192 (40.3%)	476 (100%)	
MEDIAN AGE (YEARS)			64.58± 9.1	65.36± 8.8	63.40± 8.5	64.4± 8.8	ANOVA test F=2.558. Sig. =0.078> α=0.05
DIABETES MEDICAL THERAPY			29 (25.9%)	50 (44.6%)	33 (29.5%)	112 (100%)	Pearson Chi-Square = 12.174 Asymp. Sig. (2-sided) = 0.058>α=0.05
			15 (29.4%)	16 (31.4%)	20 (39.2%)	51 (100%)	
DIABETES MIXED THERAPY			1 (16.7%)	2 (33.3%)	3 (50.0%)	6 (100%)	
ARTERIAL HYPERTENSION			165 (30.6%)	179 (33.2%)	195 (36.2%)	539 (100%)	Pearson Chi-Square = 9.705 Asymp. Sig. (2-sided) = 0.008
DYSLIPIDEMIA			123 (30.0%)	130 (31.7%)	157 (38.3%)	410 (100%)	Pearson Chi-Square = 1.424 Asymp. Sig. (2-sided) = 0.491>α=0.05
TOBACCO SMOKING			142 (31.9%)	145 (32.6%)	158 (35.5%)	445 (100%)	Pearson Chi-Square = 26.234 Asymp. Sig. (2-sided) = 0.000
CHRONIC KIDNEY DISEASE			52 (45.6%)	49 (43.0%)	13 (11.4%)	114 (100%)	Pearson Chi-Square = 40.252 Asymp. Sig. (2-sided) = 0.000
ASA GRADE 2			11(26.8%)	11(26.8%)	19 (46.3%)	41 (100%)	Pearson Chi-Square = 9.893 Asymp. Sig. (2-sided) = 0.273>α=0.05
ASA GRADE 3			99 (32.8%)	87 (28.9%)	115 (38.2%)	301 (100%)	
ASA GRADE 4			68 (29.4%)	82 (35.5%)	81 (35.1%)	231 (100%)	
ASA GRADE 5			3 (50.0%)	3 (50.0%)	0	6 (100%)	
PAD STAGE (Fontaine) 2			103 (17.8%)	65 (11.2%)	84 (14.5%)	252 (43.4%)	Value 20.636a df 4 Asymp. Sig. (2-sided) = 0.000
PAD STAGE (Fontaine) 3			44 (7.6%)	76 (13.1%)	83 (14.3%)	203 (35.0%)	
PAD STAGE (Fontaine) 4			35 (6.0%)	42 (7.2%)	48 (8.3%)	125 (21.6%)	
Total			182 (31.4%)	183 (31.6%)	215 (37.1%)	580 (100%)	

Evaluating the patency following revascularization is an important factor in comparing treatment methods in each arterial segment. Ensuring greater patency after the intervention is undoubtedly the most desired outcome of any revascularization because it reduces the likelihood of recurrence, the need for rehospitalization and reinterventions, and, most importantly, the risk of limb-related events (amputations) or cardiovascular events (cardiovascular mortality).

Patent reconstructions were reported in 536 cases, accounting for 92.4% of all studied cases. Of these, 181 cases (31.2% of all cases) were treated with EVT, 178 cases (30.7% of all patients) received OST, and 177 cases (30.5% of all patients) were treated with HST. Thrombosis was observed in 44 patients, representing 7.6% of the cohort. Among these, the highest number of thromboses occurred in 37 patients treated with OST (6.4% EVT, of all patients). Thrombosis was also present in six patients with hybrid operations (1.0% of all patients) and in one patient treated with EVT (0.2% of all patients).

The average patency of the reconstruction was investigated in days. For EVT, it is 276.12 ± 356.938 days; for hybrid surgery, it is 262.43 ± 360.060 days. For OSR, the average patency of the reconstruction is the highest at 471.74 ± 71.501 days. ANOVA testing reveals that the differences in average patency among the three treatment methods are statistically significant (F = 10.358 with a significance level Sig. = 0.000 < α = 0.05).

For patency of reconstruction, Levene's test indicates that the variances among the patient groups for the three treatment methods differ statistically (Levene statistics = 39.723 with a significance level Sig. = 0.000 < α = 0.05), which makes the results questionable. Therefore, a non-parametric Kruskal-Wallis test was additionally applied to verify the results. The Kruskal-Wallis test results do not provide a basis to conclude whether the differences in mean patency among the three treatment methods are statistically significant (chi-square = 2.578 with a significance level Asymp. Sig. = 0.275 > α = 0.05). This is due to the fact that the variances in the three groups differ statistically, and the dispersion is very large for OST.

Applying the independent samples T-test, it is found that the average patency (in days) for patients with patency is 335.43 ± 519.972 days, while for patients with thrombosis, it is 452.59 ± 597.429 days. The independent samples T-test shows that the difference of 117.164 days (about three months) between the mean patency for cases with preserved patency and cases with thrombosis is not statistically significant (t = 1.420 with a significance level Sig. (2-tailed) = 0.156 > α = 0.05).

Primary patency for EVT remained exceptionally consistent throughout the follow-up period in the first year, with only one registered case of rethrombosis. A similar distribution was observed with the hybrid group. The least number of primary patent reconstructions were found in cases subjected to OST.

Assisted primary reconstructions, identified in the analysis, were few. Across the three revascularization strategies, a total of 41 interventions were undertaken to preserve the patency of the index reconstruction. Likely due to poor patient compliance, there was insufficient detection of hemodynamically significance, yet correctable, lesions before they led to rethrombosis, making the analysis of data for secondary patency uncertain. In the OST group, interventions to preserve patency were undertaken in only four cases, and that was six or more months after the bypass construction. In HST and EVT, 22 and 15 cases were observed, respectively. In the EVT group, a larger number of restenoses were discovered and intervened upon in the third month - five cases, with a relative share of 2.7%. In the same group, another peak in the discovery of restenotic complications was observed after the end of the first year. A gradual progression in the detection and treatment of stenotic complications over time was observed in the HST cases.

When considering data for reconstructions that underwent subsequent intervention to restore patency due to rethrombosis, the following trends were observed. Within the first month after reconstruction, thrombotic complications were recorded twice as frequently in the hybrid and open groups compared to endovascularly treated patients. For OST, there is a decrease in the need for reintervention due to thrombotic complications by the end of the first year, with such reinterventions reappearing in the 12th month and thereafter. In the EVT and HST groups, no significant differences in the frequency of rethrombosis were observed, nor indirectly in the need for intervention to preserve patency. Only after the first year, a 50% increase in these interventions was observed in hybrid-treated patients compared to the endovascular group and 25% more interventions for restoring patency compared to the conventional OST.

**Figure 1:**
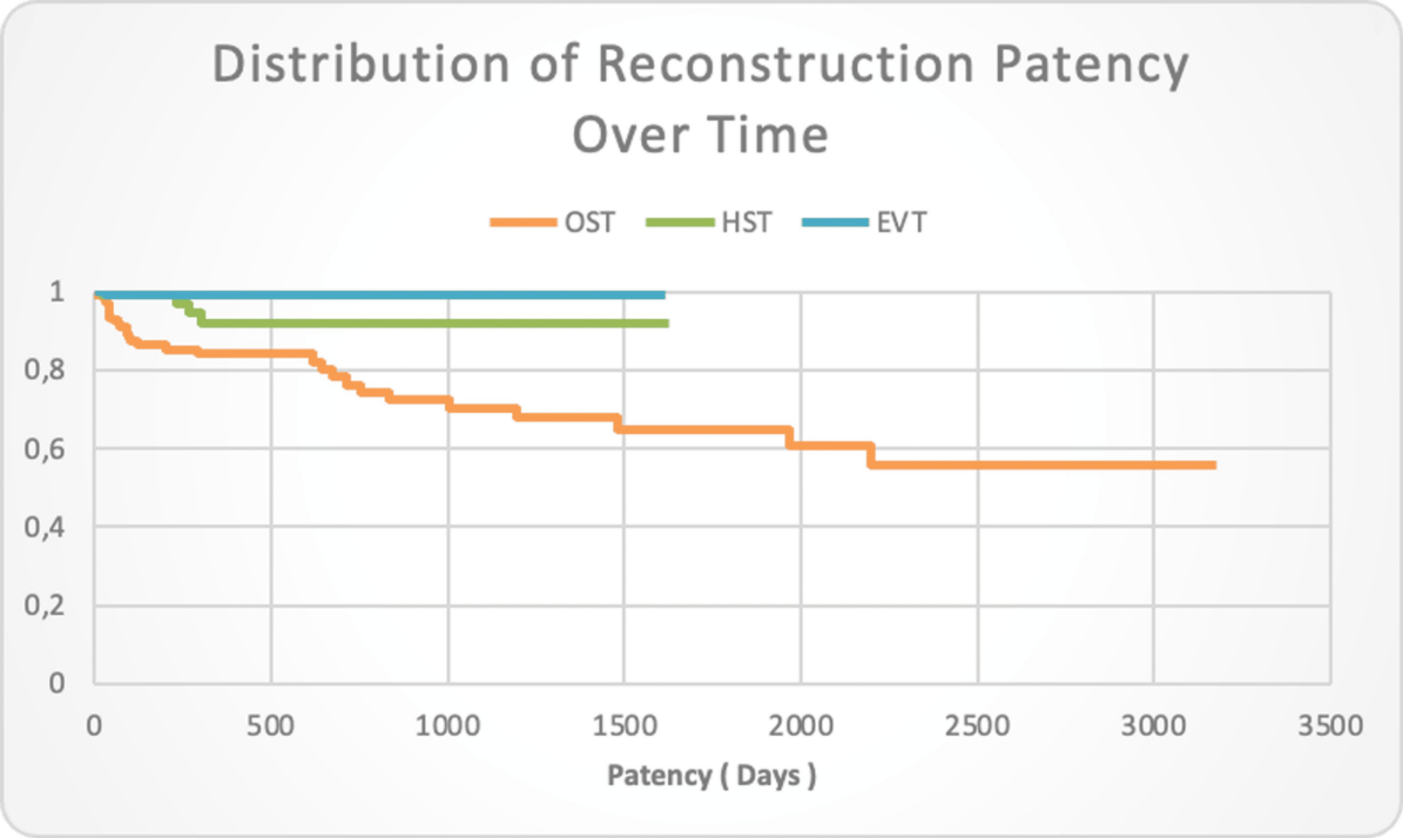
Distribution of reconstruction patency over time in ОST, HST, and EVT with confidence interval EVT-Endovascular treatment; HST-Hybrid surgical treatment; ОST-open surgical treatment

The average time for maintaining patency of the reconstruction with OST was calculated to be 1904.6 days (with SD of 130.8 days). The 95% confidence interval (CI) indicates that we can confidently expect the average reconstruction survival time to be between 1,648 and 2,160 days (approximately 4.5-5.9 years). The average survival time of reconstructions in patients undergoing hybrid treatment was calculated to be 1,471.6 days (SD: 63.6 days). The 95% CI indicates that we can confidently expect the average survival time of hybrid reconstructions to be between 1,347.0 and 1,596.2 days (approximately 3.6-4.4 years). When interpreting these data, it should be considered that, in the present retrospective study, endovascular and hybrid procedures have been followed for a much shorter period (five years). In the endovascular group, the average survival time of the reconstruction is 981 days. The studied group registered only one thrombotic complication, significantly narrowing the confidence interval and, accordingly, bringing the probability of maintaining patency of the reconstruction above 95% for a follow-up period of five years (Figure 1).

Complications in the Three Revascularization Methods

Complications associated with surgical treatment for TASC II C-D lesions in the aorto-iliac segment were considered more serious compared to those in EVT and HST. This is due to the greater surgical trauma involved, the use of more synthetic material, and the application of general anesthesia. Complications observed in patients who underwent surgical treatment are presented in Table 2.

**Table 2:** Complications in the three different revascularization strategies EVT-Endovascular treatment; HST-Hybrid surgical treatment; OST-Open surgical treatment

	Type of revascularization			
Complication	EVT	HST	OST	Total
Infection	0	6	19	25
	0.00%	1.00%	3.30%	4.30%
Rethrombosis	2	6	36	44
	0.30%	1.00%	6.20%	7.60%
Bleeding	0	0	2	2
	0.00%	0.00%	0.30%	0.30%
Pseudoaneurysm	1	0	1	2
	0.20%	0.00%	0.20%	0.30%
Anastomotic stenosis	0	4	6	10
	0.00%	0.70%	1.00%	1.70%
Puncture site complication	4	1	0	5
	0.70%	0.20%	0.00%	1.00%
Ileus	0	0	1	1
	0.00%	0.00%	0.20%	0.20%
Limb amputation	4	6	12	22
	0.70%	1.00%	2.10%	3.80%
Death	0	0	5	5
	0.00%	0.00%	0.90%	0.90%
% of Total				

Due to its minimally invasive nature and the use of only local anesthesia, EVT for PAD is associated with significantly fewer complications. In the current analysis, early perioperative mortality was recorded only in the OST group in five clinical cases, which amounts to 2.3% of the group and 0.86% of the entire studied sample. One case involved a myocardial infarction, two cases of arrhythmic death, one case of multi-organ failure, and one case of sepsis. Mortality related to cardiovascular events amounted to three cases - 1.39% of the OST group and 0.51% of the studied population. Perioperative mortality was not recorded in EVT and HST. Local complications, such as hemorrhage from the reconstruction, were not found in EVT and HST, while two bleeding complications occurred in the OST group, requiring surgical revision for hemostasis. Hematomas at the puncture site, also leading to surgical revision, were found in four endovascular cases and in one in the HST group. Among all 580 studied cases of revascularization in the aorto-iliac segment, complications such as lymphatic cysts and neurological damage were not observed.

Impact of PAD Clinical Presentation on the Patency of Reconstructions Among the Three Methods

The impact of PAD's clinical presentation - claudication and CLTI - on the patency of reconstructions was analyzed. According to the Fontaine classification, it is assumed that patients in the first and second stages present with claudication, while cases in the third and fourth stages have a clinical presentation of CLTI.

**Figure 2:**
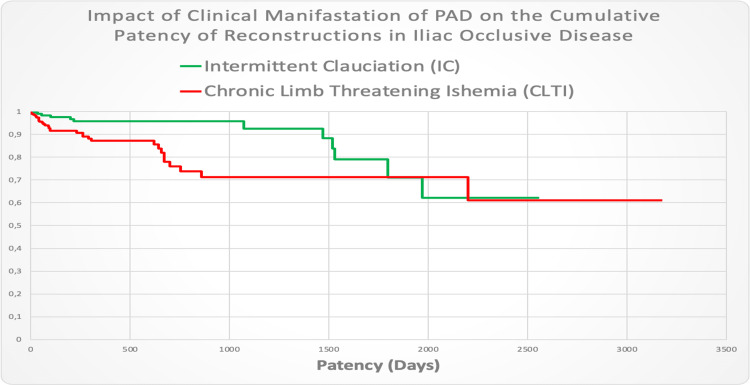
Impact of clinical manifestation of PAD on the cumulative patency of reconstructions in iliac occlusive disease

In examining the influence of the PAD stage - through its clinical presentation of claudication - on the patency of reconstructions undertaken, it was found that the average survival time of reconstruction patency was 2,099.6 days (SD of 105.196). The results from the analyzed data indicate that undertaking any revascularization of the iliac segment carries a 50% risk of losing patency after the fifth year. In the next examined subpopulation with a clinical manifestation of CLTI, the average survival time of the reconstruction was shorter - 1,965 days (SD with a standard deviation of 119.8 days; Figure 2).

In cases of CLTI, the probability of losing patency is higher and occurs earlier. For the claudicant group, the loss of patency was below 5% and gradually decreased within the third year. The probability of losing patency of the iliac reconstruction becomes equal between the two examined groups where the two curves overlap after the five-year period.

The log-rank test shows an observed value of 9.021 with a critical value of 3.841 (p = 0.003 < alpha = 0.050). This indicates the presence of statistically significant differences in the survival distribution of reconstruction patency between the groups of claudicants and the cases with CLTI.

**Figure 3:**
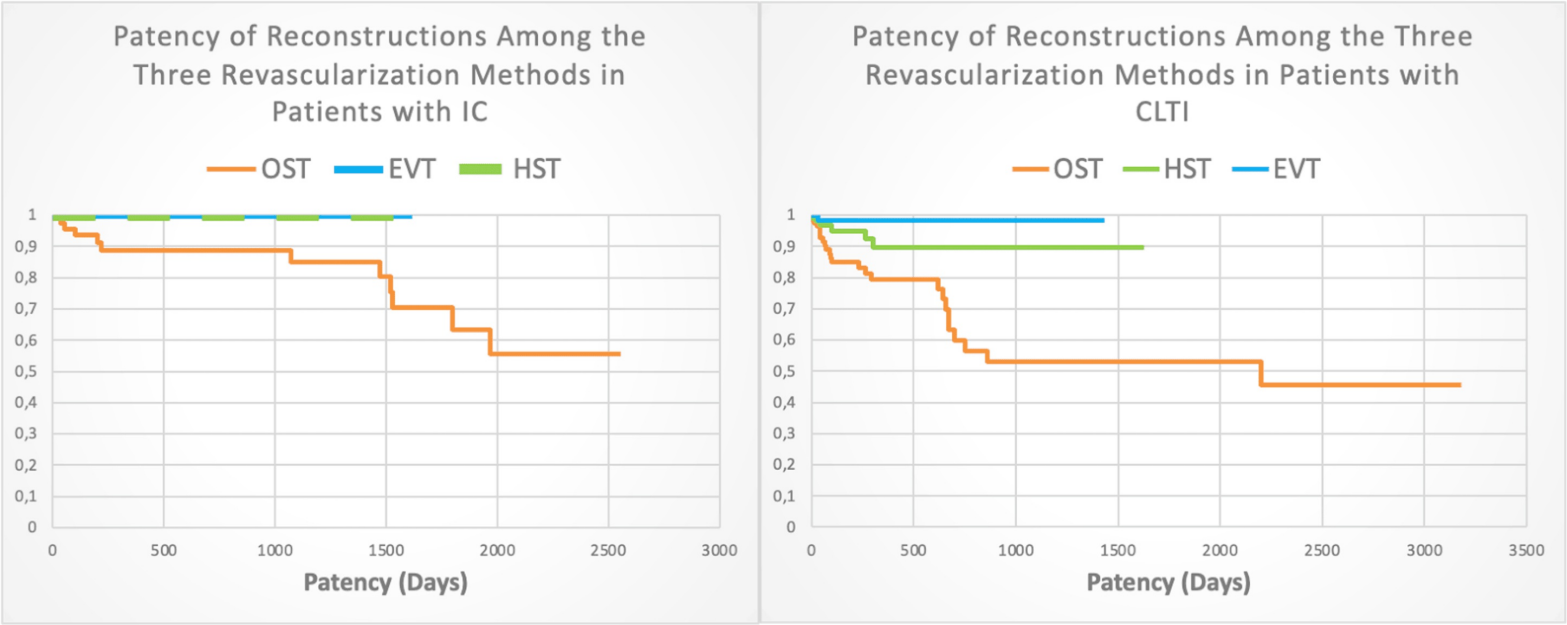
Analysis of the patency of reconstructions among the three revascularization methods in patients with claudication manifestation of PAD and CLTI EVT-Endovascular treatment; HST-Hybrid surgical treatment; OST-Open surgical treatment

The next part of the analysis examined the impact of the two clinical presentations of PAD - claudication and CLTI - on the patency of reconstructions among the three compared revascularization methods (Figure 3).

Upon applying the log-rank test, a p-value of 0.005 was found. This indicates the presence of statistically significant differences in the survival of reconstructions among the studied groups with different revascularization strategies. The comparative analysis revealed a statistically significant difference between the groups receiving OST and EVT (p = 0.008 < 0.05), as well as between the OST and HST groups (p = 0.039 < 0.05). There were no statistically significant differences between the EVT and HST groups, with a p-value above 0.05 (p = 1.000).

The comparisons show statistical significance between the OST versus EVT groups and the OST versus HST groups, but not between EVT and HST. In the subgroup of patients with claudication, no differences in the patency of the reconstruction over time were expected if the applied method is endovascular or hybrid. The surgical approach as a choice of revascularization strategy in this subgroup demonstrated a reduction in patency by about 10% already in the first year.

The log-rank test indicates that the observed value was significantly higher than the critical value, resulting in a very small p-value (0.000). This means that there were statistically significant differences in survival between the groups, leading to the rejection of the null hypothesis for the absence of survival differences among the groups subjected to different revascularization strategies.

When conducting multiple comparisons, statistically significant differences were found between the groups, with the adjusted p-value (Dunn-Sidak) being below 0.05 (0.019) for open versus hybrid treatment. Similar findings are observed in the comparison between OST and EVT groups, with a p-value of 0.002 being below 0.05 (0.005).

In summary, the comparisons show statistical significance between the OST and HST groups, and OST and EVT groups, but not between HST and EVT.

In the subgroup of patients with CLTI, there was no difference in the patency of endovascular and hybrid reconstructions over time, and it remains relatively high with an advantage in endovascular treatment. However, in patients with CLTI who underwent open surgery, a statistically significant difference and a higher risk of losing patency of the reconstruction earlier in time were observed.

Primary Patency Rates of the Three Treatment Methods for the Entire Population Studied Over a Period of One Year and Beyond

After the statistical processing of data from the current retrospective study, patency of the reconstructions was recorded in 536 cases, constituting 92.4% of all assessed events. Of these, 181 (33.8% or 31.2% of all cases) were with EVT, 178 (33.2% or 30.7% of all cases) were with OST, and 177 (33.0% or 30.5% of all cases) were with HST. Rethrombosis was found in 44 cases, accounting for 7.6%. Of these, the highest number of rethromboses occurred in 37 cases treated with OST (84.1% or 6.4% of all cases). Rethrombosis also occurred in 6 hybrid surgical interventions (13.6% or 1.0% of all cases) and in 1 patient with EVT (2.3% or 0.2% of all patients). The χ2 test shows a statistically significant relationship between the patency of the reconstruction and the choice of treatment method (Pearson χ2= 46.097 with a significance level of Asymp. Sig. (2-sided) = 0.000 < α = 0.05).

**Figure 4:**
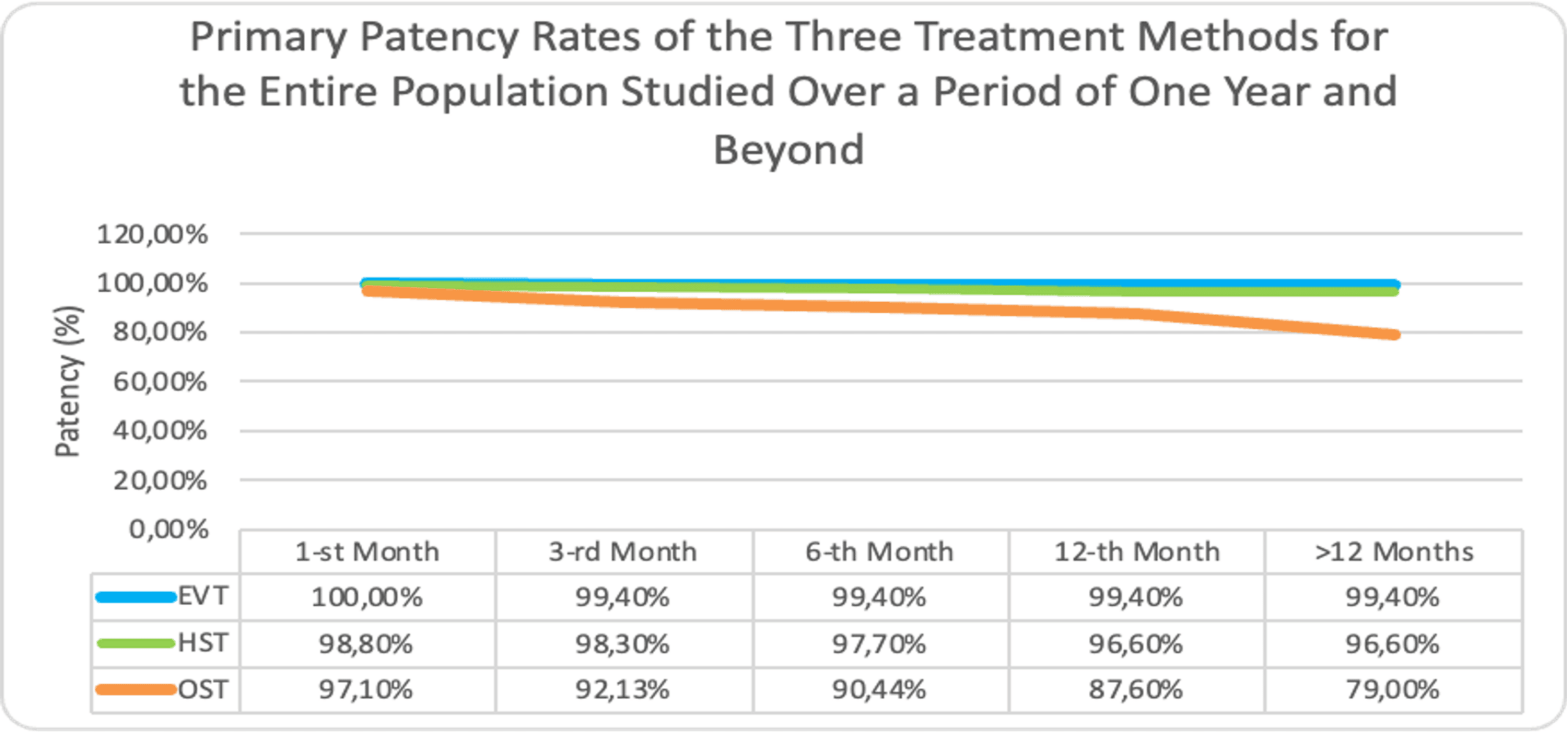
Primary patency rates of the three treatment methods for the entire population studied over a period of one year and beyond EVT-Endovascular treatment; HST-Hybrid surgical treatment; OST-Open surgical treatment

When considered, over time, the patency of the three types of reconstructions in 148 patients followed for the first postoperative month, 141 (95.3%) had patent reconstructions, and only seven (4.7%) were recorded with early rethrombosis. Of the patients with patent reconstructions, 49 (34.8% or 33.1% of all followed during this period) were with OST, 46 (each 32.6% or 31.1% of all followed during this period) were with EVT, and the same number were with HST (Figure 4). This trend continued in the subsequent months up to the first year, where it is notable that, in the EVT group, there was a predominance of patent reconstructions by 15.1% compared to hybrid surgery and 11.4% compared to classical open treatment. However, in the long term, by the fifth year of follow-up, the lead returned to open treatment, where twice as many patent reconstructions were registered.

The graphic illustrates that, within the first month, the patency rates remain very high across all three methodologies, but with a preference for endovascular treatment (with the greatest difference of 3% compared to surgical treatment). However, by the end of the first year, the patency rates decrease significantly with the surgical approach and to a lesser extent with hybrid treatment. This outlined difference in favor of the endovascular method amounts to 2.8% compared to hybrid and 11.8% when compared to open surgery. Based on these data, it can be concluded that endovascular and hybrid treatments demonstrated comparable frequencies of primary patency within a one-year follow-up period. In the latter part, the curve for OST sharply declined due to a larger number of patients being followed for a longer period of time, inevitably leading to an increase in the frequency of registered rethromboses. Patient follow-up compliance with the recommended follow-up schedule can also influence the observed trends.

Primary-Assisted Patency Reconstructions for the Three Revascularization Methods Followed for a Period up to One Year and Beyond

**Figure 5:**
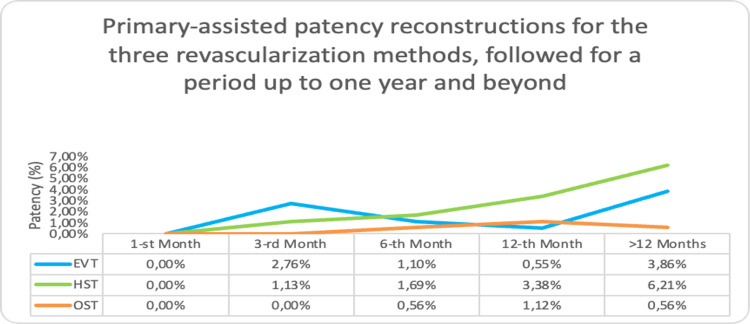
Primary-assisted patency reconstructions for the three revascularization methods, followed for a period of up to one year and beyond EVT-Endovascular treatment; HST-Hybrid surgical treatment; OST-Open surgical treatment

In comparison to primary patency, which shows a smooth decline across all three treatment methods, primary-assisted patency demonstrated distinct trends within a one-year follow-up period, as illustrated in Figure 5. EVT exhibited a steep increase in patency during the third month, followed by a reduced need for patency-assisting reintervention through the first year. A surge in restenosis was diagnosed, and preventive treatment occurred after the 12th month. With hybrid treatment, the incidence of restenosis and its treatment steadily rises. For open treatment, these events peak within the first year after a slow buildup.

Secondary patency rates are similarly high across all revascularization strategies. Endovascular treatment reported a 14.3% reduction in patency by the end of the first year. Open treatment achieved an 86.4% secondary patency rate at the same mark, with a 13.6% loss in patency. The hybrid surgery group maintained a high patency until the third month and then dropped to 14.2% by the sixth month - earlier than both endovascular and open treatments. Nevertheless, at the first postoperative year's end, bypass surgery showed the highest secondary patency (at 86.4%).

Cumulative Patency Across the Three Treatment Methods

**Figure 6:**
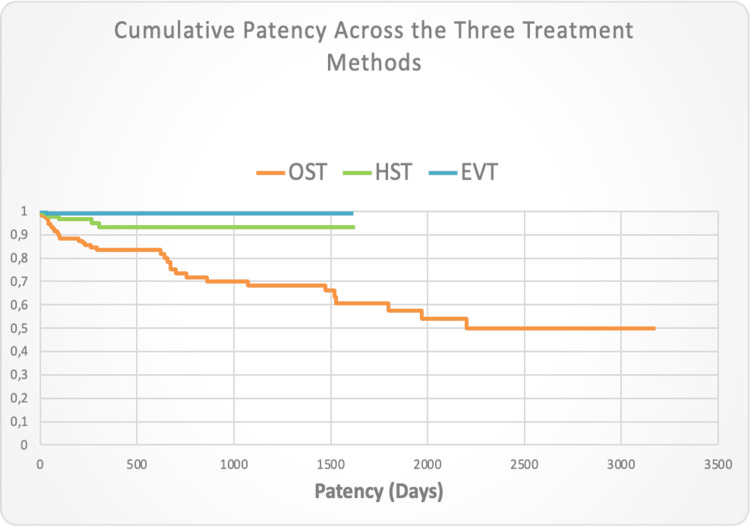
Cumulative patency across the three treatment methods EVT-Endovascular treatment; HST-Hybrid surgical treatment; OST-Open surgical treatment

Figure 6 shows the cumulative patency rates for the three revascularization strategies throughout the follow-up period, revealing significant differences through equality tests (log-rank, Wilcoxon, Tarone-Ware) with log-rank test highlighting significant disparities (χ² = 27.943, p < 0.0001). The comparison of methods (OST vs. HST and OST vs. EVT) revealed significant differences, indicating lower cumulative patency for OST compared to both HST and EVT, underscoring the critical role of method selection on patency outcomes. The HST vs. EVT comparison, while not statistically significant (p = 0.053, adjusted p = 0.152), suggested a potential trend that could be meaningful with more data.

## Discussion

Indications for Surgical Treatment

At the start of the 21st century, the leading factor in choosing a treatment approach was the anatomical classification of the lesion, TASC II, issued in 2007 as an updated version of its first edition from 2000. Currently, all practitioners involved in vascular medicine agree that lesion the anatomical pattern is entirely insufficient to be the sole reference point for an informed decision on the treatment method [1]. PAD is a disease that often affects various segments of the arterial tree. It is taken as a fundamental truth that the revascularization of the lower limb, regardless of the clinical presentation of the disease, starts with ensuring adequate antegrade blood flow in the aorto-ilio-femoral segment, regardless of the presence of simultaneous underlying involvement.

Current guidelines state that the first choice of treatment for patients with claudication is always conservative treatment, lifestyle modification, and supervised exercise therapy. The American College of Cardiology and the American Heart Association in 2016 published their guidelines for the diagnosis and treatment of PAD. At present, this document is the main source providing evidence-based recommendations for managing patients with claudication. It specifies that endovascular procedures are effective as a revascularization option for patients with claudication that limits their lifestyle and who have hemodynamically significant aorto-iliac occlusive disease. The level of evidence for this recommendation is I A [1].

Several systematic reviews have published conclusions that surgical procedures are an effective treatment for patients exhibiting symptoms of intermittent claudication and have a positive impact on their quality of life and walking parameters (speed, pain-free walking distance). However, these studies find scant evidence supporting the efficacy of surgery compared to other alternative treatments [2,4,5]. Improvements in symptoms and patency of reconstructions with OST could be superior compared to less invasive endovascular techniques. Nonetheless, open surgical interventions are inevitably associated with a higher risk of adverse perioperative events. The choice of the most appropriate surgical treatment should be individualized based on the objectives we aim to achieve for the patient, the perioperative risk, and the expected benefit [6].

Surgical interventions with an open approach in patients with claudication are indicated for individuals who 1) have not gained sufficient/adequate benefit from non-surgical treatment and optimal medical therapy (OMT), 2) have arterial anatomy favorable for conducting open surgery, and 3) have an acceptable risk of perioperative adverse events [1].

In 2019, the Global Vascular Guidelines on the Management of CLTI were published [7]. In this internationally recognized guide, inflow disease is defined as hemodynamically significant stenosis or occlusion proximal to the origin of the superficial femoral artery, meeting one or more of the following criteria: 1) absence of femoral pulse; 2) dampened pulse waveform, assessed at the level of the ankle-brachial index (ABI) during Doppler ultrasound examination; 3) presence of >50% stenosis in the aorto-iliac segment or ABI, proven through angiography; and 4) measured systolic pressure gradient from the aorta to ABI >10 mmHg at rest. If the patient presents with rest pain and minor tissue loss in the foot area, correction of the flow in the iliac segment may be sufficient to achieve the desired clinical outcome. As the complexity of the planned procedure increases, indicators such as perioperative morbidity and mortality also rise. Most aorto-iliac disease patterns can be successfully treated with endovascular approaches and the implantation of appropriate stents [8].

Open surgery is often reserved for long-segment iliac occlusions or after the failure of a previously conducted endovascular procedure. The choice of an OST to ensure adequate antegrade blood flow should be based on a combination of factors such as the patient's potential risk, the anatomical pattern of the disease, and other clinical factors. An anatomical bypass (e.g., aorto-femoral/ilio-femoral bypass) is always preferable over any extra-anatomical reconstruction. Recommendation 6.20 states that, for patients with CLTI and simultaneous involvement of the iliac and femoropopliteal segment, iliac reconstruction precedes the distal one with a level of evidence - I C. Recommendation 6.25 suggests the application of the endovascular approach as the first choice in patients with CLTI with moderate to severe disease manifestation (GLASS stage I A), involvement of the aorto-iliac segment, and medical history of previous intervention. The level of evidence for this recommendation is 1B. Recommendation 6.26, with a level of evidence 2C, states that surgical treatment is appropriate for a patient with CLTI, falling into the moderate-risk group with proven extensive aorto-iliac involvement (GLASS stage 2) or after a failed previous endovascular procedure [1].

In a meta-analysis by Premaratne et al., direct surgical versus endovascular revascularization for IOD was compared. The authors found no statistically significant differences in the main characteristics of patients between the two groups: gender, stage of PAD manifested with claudication, presence of rest pain, tissue loss from the distal part of the limb, preoperative ABI, and the anatomical type of lesion TASC C and D [9].

In our representative sample, a statistically significant relationship was observed between the choice of treatment method and some of the main patient characteristics: gender, diabetes, chronic kidney disease, arterial hypertension, smoking, and anesthesiological risk (see Table 1).

When examining the PAD stage as a criterion for choosing a revascularization method, the χ2-test shows a statistically significant relationship between PAD stages and the applied treatment method (Pearson χ2 = 20.636 with significance level Asymp. Sig. (2-sided) = 0.000 < α = 0.05). Most patients in Fontaine stage 2 (with claudication) underwent EVT, 103 (40.9%), and most of the patients in stages 3 (rest pain) and 4 (tissue loss) underwent OST and HST. The calculation of Cramer's V coefficient = 0.133 is statistically significant with a significance level Approx. Sig. = 0.000 < α = 0.05. The value of the coefficient is below 0.3, indicating a weak correlation between PAD stages and the choice of treatment method. However, patients in the second stage of the disease are predominantly treated through the endovascular surgical approach, in full accordance with global trends and recommendations for the primary application of minimally invasive techniques. The open and hybrid surgical approaches significantly prevail among patients in PAD stages 3 and 4. The choice of a hybrid strategy for iliac revascularization is usually associated with the presence of hemodynamically significant occlusion of the target artery - most often CFA, deep femoral artery (DFA), superficial femoral artery (SFA), or spread of PAD in underlying segments, which is directly proportional to the advancement of the disease stage.

The data from the comparative analysis proved that, over time, EVT has become the first choice, especially in patients in earlier stages of the disease, while the more traumatic surgical treatment is chosen in advanced stages of IOD, explained by the extensive spread of the atherosclerotic process in the vessels of the lower limb. Regarding the hybrid surgical approach, the disease stage is not the leading criterion for choice, but rather concomitant diseases and the involvement of the CFA by the atherosclerotic process. Although numerous comparative analyses of results from endovascular and open surgical treatment in various arterial segments have been published, the scientific world needs prospective studies linking the PAD stage with the choice of the surgical approach in IOD.

The goal of invasive treatment in patients with iliac involvement from PAD is to provide mainline revascularization of the infrainguinal arterial segments and improve circulation to the tissues of the lower limb. Besides technical success, revascularization aims to achieve other outcomes depending on the specific groups of patients with PAD. For example, in claudicants, the goal is to improve symptoms, increase pain-free walking distance, and have an overall positive impact on their quality of life. For patients with CLTI, invasive treatment should lead to limb salvage, healing of distal ischemic lesions, quality of life improvement, and reduction in overall and cardiovascular mortality. Technical success of revascularization in the context of the proposed analysis is accounted for by the restoration of the femoral pulse post-procedure. In the current analysis, the overall technical success across the three types of reconstructions amounts to 98%, with no statistically significant difference found between the individual techniques. Various authors report a technical success rate of the procedure (surgical, endovascular, hybrid) over 90%, and in some cases, even up to 100% [10,11].

Objective criteria for evaluating revascularization include the following: 1) the absence of the need for repeat revascularization (reinterventions) and 2) limb preservation (the number of major amputations performed).

Data show that, despite comparable post-procedural results, the three methodologies provide different patency rates of reconstructions over time. Revascularization aimed at maintaining primary assisted patency (conducting invasive treatment due to detected restenoses) is most represented in the hybrid treatment group (12.02% of all hybrid interventions), followed by EVT (8.2% of all reconstructions in this group). In the surgical treatment group, only four reconstructions were primarily assisted, with a relative share of 1.8% of all open surgeries. Regarding additional revascularization undertaken to ensure secondary patency (after a proven rethrombosis of the reconstruction), the hybrid surgical approach is again in the lead (6.55%), followed by endovascular treatment and open surgery (5.5% and 4.2%, respectively). The cumulative need for revascularization is to preserve the reconstruction as determined by the hybrid strategy (18.6%), followed by endovascular treatment (13.7%, both accounting for preventatively intervened restenoses). The least frequently undertaken intervention to maintain patency is seen in conventional open surgery (6% of cases).

Zavatta et al. in 2017 published a large cohort study of 879 patients, comparing the results of OST and HST in IOD with peri-procedural complications, follow-up of clinical indicators, improvement in ABI, and the patients' ambulatory functional status. Their research adds information to other studies reporting 75-97% primary patency and 95-98% secondary patency in the third and fifth year after HST, respectively [12].

The limb salvage rate in the examined population was high and comparable across all three revascularization strategies. The highest percentage of limb preservation was recorded in the EVT group (97.8%), followed by the hybrid technique (96.7%), and the lowest frequency of limb salvage was demonstrated by OST (94.4%). Endovascular interventions focus on minimally invasive procedures to restore blood flow to the lower limb. Chen et al. reported that endovascular revascularization alone as an approach can provide satisfactory distal arterial blood flow, sufficient for limb salvage [13].

This is confirmed by the results of the current analysis. Hybrid interventions, combining endovascular and surgical techniques, have become an integral part of the vascular surgeon's arsenal in the fight to save limbs threatened by amputation [14]. Studies by Huynh et al. highlighted their importance in patients with multi-level arterial occlusive disease, demonstrating positive results in terms of limb salvage rates after EVT in the first and second year, amounting to 98.9% and 98.4%, respectively [14]. Piazza et al. reported similar excellent outcomes from their experience at the Mayo Clinic. The authors show no differences in three-year patency and limb salvage rates, comparing OST with a hybrid approach and stenting of the iliac artery ( 91% versus 97%, respectively) and conventional femoral endarterectomy (100% versus 100%, respectively) in their study of 92 patients [11].

Patency of Reconstructions

The technical execution of any revascularization strategy inevitably affects both the survival of the reconstruction and the final outcome for the patient. Not every rethrombosis that occurs in the revascularized segment will lead to limb loss. However, the patency of the reconstruction is a predictor of the risk of amputation and relates to the recurrence of the patient's complaints, subsequent hospitalizations, and reinterventions. In open surgical treatment, factors such as the choice of sites for the proximal and distal anastomoses of the bypass, the size/diameter of the graft, the presence of a good outlet to the deep and superficial femoral artery, and the execution of the anastomoses are crucial for treatment success. With contemporary endovascular approaches and their combination with classical open techniques, the variability of these factors increases significantly, further complicating analyses and achieving statistically verified conclusions. A systematic analysis of six studies from 1995 to 2016 describes 532 hybrid procedures [1].

All studies included in this analysis are retrospective and non-randomized. Five of them have been assessed as having low methodological quality, with the exception of the study. Several different techniques of remote endarterectomy are described, which are associated with better patency of the reconstructions. Bekken et al. used a balloon for angioplasty before placing the ring stripper to ensure a dissection plane [15]. Smeets et al.'s [16] is the exception as this is the only study with a standardized protocol for postoperative patient follow-up. Kavanagh et al. used a balloon in the stripper for the dissection of the intimal cast instead of the specialized MollRing Cutter [17]. In all studies, except that of Smeets et al., the primary lesion is overcome with a guidewire, over which the stripper is placed. Simo et al. directly stented the proximally transected segment, while other studies describe a selective stenting strategy - only for residual high-grade stenosis or flow-limiting dissection [18].

The frequency of stent implantation in these studies varies widely from 11.1% to 72.6%. The surgical techniques used in the current analysis are described in detail, but no data are collected regarding the aforementioned specifics in hybrid and endovascular surgical treatment. In the published studies, stenting of the intervened segment is optional and has various frequencies. In the presented study, the cases were selected so that all iliac lesions have undergone stent implantation after their endovascular revascularization and in hybrid intervened cases (64%).

The definition of patency for a reconstruction varies significantly in the available publications. Simo et al. considered that clinical success and patency are determined by the disease stage according to the Rutherford classification, and only thrombosis of the reconstruction is regarded as a loss of patency. Smeets et al. provided their definition of patency as the absence of occlusion or >70% restenosis. Kavanagh et al. and Topel et al. did not provide a definition of what they considered a patent reconstruction [16-19].

In the most recently published large studies, there is a shift from the term "patency," which is considered primarily a technical characteristic of the reconstruction and not a predictor of the final outcome for the patient. In the literature, along with the frequency of reinterventions, amputation, and mortality, concepts such as "amputation-free survival" and "overall survival" are increasingly reported, which are much more oriented towards the effect on the patient and their quality of life [16-19]. The term "freedom from target lesion revascularization" (freedom from TLR) is used as a synonym for patency. However, this concept implies that the patient is not a candidate for subsequent invasive treatment due to a compensated condition of their limb, which does not mean that the reconstructed segment is patent. Some authors consider that patency is an indicator that should be evaluated, especially in cases where we compare alternative surgical techniques and seek the most suitable one to build a therapeutic algorithm.

Choosing the right revascularization strategy is crucial for the outcome and follow-up events post-thrombosis. Endovascular approaches have fewer thrombotic events in the initial month but experience an uptick thereafter. Conversely, open surgical methods, such as bypass, lead to prolonged event occurrences. Beyond the first year, an increased frequency of rethrombosis and the necessity for interventions to preserve patency are observed across all revascularization strategies (Figure 5).

Open surgery is the gold standard for PAD treatment, providing excellent long-term patency but with perioperative risks [12].

In our representative sample, patency rates for 6-12-month follow-up for patients treated with HST were 90.5%, aligning with aggregated patency from various studies. Other reports on primary-assisted patency revealed rates of 90.8% in the first year, 86.6% by the third, and 81.7% by the fifth year, with secondary patency rates at 92.7%, 89.9%, and 84.7%, respectively. The overall technical success for reconstructions averaged 90% in all those studies. In the presented sample, primary patency was achieved in 92.4%, distributed nearly evenly across endovascular, open, and hybrid treatments.

Two groups have reported on the clinical success of reconstructions. Kavanagh's group reported clinical improvements in all patients post-surgery [17]. Simo et al. defined success as an ABI increase >0.1 and at least one Rutherford stage improvement, achieving a 76.6% success rate [18]. Additionally, 18.6% improved in ABI but not in the Rutherford stage, while 3.4% with no ABI change. Clinical worsening occurred in 1.3% of the cases, resulting in unexpected major amputations.

Bekken et al.'s 2018 study on AIOD patients revealed that hybrid surgery matches open surgery in technical success and limb salvage for high-risk patients, without increasing complication risks. Hybrid surgeries showed slightly lower primary patency than open surgeries, with rates of 87.0%, 75.5%, and 69.2% over three years, and no 30-day mortality was reported [15]. Chen et al. in the same year advocated for endovascular and hybrid approaches for occlusive iliac lesions, noting the hybrid technique's efficacy in enhancing distal blood flow and simplifying endovascular procedures for complex multilevel lesions [13]. The retrospective study highlighted superior primary patency in open and hybrid surgeries at 87.6% and 96.6% for the first year, underscoring their effectiveness in treating atherosclerotic aortoiliac occlusive diseases (AIOD).

Technical failures in the procedure primarily arise from challenges in advancing the ring stripper through the subintimal plane and adventitial injuries leading to arterial perforation, risking significant and rapid blood loss. Routinely using a guidewire for lesion crossing in this case series improves procedure safety and lowers the risk of severe complications, such as iliac artery injury. Additionally, the guidewire aids in navigating the ring stripper with fluoroscopic guidance and allows for quick stent graft implantation when needed, thereby preventing the shift to more invasive surgical methods and the creation of an ilio-femoral bypass.

The hybrid surgical approach boasts notably low morbidity and mortality (0.4%, 30-day rate), outperforming bypass surgery, which has a 30% complication rate and 3% mortality [15].

This comparative analysis shows a higher major amputation rate in open surgery patients at 2.1%, compared to 1% in hybrid group patients, with no deaths reported in hybrid cases and a 0.9% mortality rate in conventional surgeries.

The most common complications after HST are minor, primarily related to the surgical wound, and can be successfully treated with conservative measures. Compared to aorto-femoral and ilio-femoral bypass, HST is a less invasive technique that avoids abdominal or retroperitoneal surgical access and aortic clamping. These advantages make it suitable for patients with severe cardiovascular and pulmonary diseases, who would otherwise be considered high-risk for surgical revascularization. Surgeons often prefer ilio-femoral over aorto-femoral bypass for patients with a patent CIA, to avoid midline laparotomy and aortic clamping. However, even the retroperitoneal approach involves more surgical trauma than a single femoral incision. In a major review comparing open to endovascular treatment of aorto-iliac occlusive disease, five-year patency was estimated at 82.7% for primary and 91.0% for secondary patency [20].

The primary patency rate for the HST group was 98.80% at one month and 96.6% at 12 months and long term, whereas in the bypass surgery group, it was 97.7% at one month and 87.6% at 12 months and dropped to 79% at five years. This decline in patency rates for open treatment could be due to better adherence to long-term follow-up and more consistent complication detection compared to the other groups. The greater surgical trauma likely has a psychological impact on the patient, encouraging them to be more engaged with the long-term outcome of the reconstruction.

Indes's meta-analysis, covering 29 open surgery and 28 endovascular studies, revealed that open surgery patients experienced more complications (2.6% vs. 0.7%; p = 0.001) and higher 30-day mortality (18.0% vs. 13.4%; p = 0.001) than those undergoing endovascular treatments [21,22]. These differences likely reflect the vulnerability and comorbidity of open-surgery patients. Our study's findings align, showing a 0.9% mortality rate, all within the open surgery group, markedly surpassing the literature's reported figures. Deaths predominantly involved patients with higher ASA classifications and anesthesiological risk due to extensive comorbidities and deteriorated health.

Carsten et al. reported a five-year secondary patency of 93.3%. In comparison, the HST group in this study showed higher patency rates [22].

Restenosis after HST typically stays asymptomatic, possibly due to patients having preoperative total occlusions and a robust collateral network that remains unaffected post-intervention. Comorbidities might limit patients' physical activity, reducing the likelihood of symptom emergence. Therefore, without an active search for a vascular surgical assessment, patency loss might go unnoticed.

Research showed lower patency for EIA stenting versus the CIA [16]. The Covered vs Balloon Expandable Stent Trial (COBEST) trial found a five-year patency of 73.9% for covered stents and 62.9% for uncovered stents in these arteries [23]. Bekken et al. highlighted endovascular treatment's lower patency, particularly for lesions affecting the CFA, with only a 45% two-year assisted patency for combined lesions [15]. Contrary to these findings, our analysis revealed similar high one-year secondary patency rates across all revascularization methods: 85.7% for endovascular, 86.4% for open surgery, and 85.8% for hybrid surgery, indicating effective outcomes across different strategies.

While endovascular treatment complications are under 10%, for severe CFA atherosclerosis, surgeons prefer endarterectomy to angioplasty [24]. A study showed a 22% complication rate and 60% five-year primary patency for patients undergoing femoral desobliteration and iliac stenting. Patients progressing from external to CFA involvement are ideal for HST, balancing improved patency with manageable morbidity [15]. Stenting strategies differ, with selective stenting being common. In our study, all endovascular patients received stents due to lesion complexity and CIA involvement. Hybrid surgery required stents in 64% of cases, as not all endarterectomies reach clear CIA sections, sometimes requiring angioplasty [15]. Simo et al. suggested primary stenting at transection sites, with restenosis often near proximal cuts [19]. Our findings showed no patency differences between stented and non-stented proximal segments, highlighting the need to further explore optimal stenting strategies for HST.

Piazza et al.'s 2011 retrospective study reviewed 92 patients treated with OST and 70 with HST and found comparable three-year primary patency rates between the groups. Stratification by TASC lesion categories revealed significant tissue loss as a predictor of decreased long-term patency for hybrid interventions, though the study did not include functional outcome data [25].

This study predicts reduced patency in reconstructions as the disease progresses to CLTI and with tissue loss, with Kaplan-Meier analysis highlighting greater patency loss in open and then hybrid reconstructions, whereas endovascular treatments fare best in patency rates. Zavatta et al. further confirmed similar patency and mortality outcomes, emphasizing that OST yields superior functional results [12].

Improvements in walking capacity seen in the OST group versus HST might not be unique to inflow PAD treatment and could extend to infrainguinal disease cases. Zhou et al.'s 2014 study involving 64 OST and 43 HST patients for infrainguinal occlusive disease found that, with 85% treated for CLTI, there were equivalent increases in ABI and Rutherford stage improvements between groups, with no significant patency differences at 36 months, suggesting comparable outcomes across treatment strategies [26].

The observations discussed reflect a paradigm shift in treatment over the last decade, with an increase in the use of endovascular and hybrid techniques as the first line of treatment, even for extensive lesions traditionally managed with open surgical intervention. This shift underscores the evolving nature of medical practices, favoring less invasive approaches where possible to optimize patient outcomes and recovery times [25,27-29].

Complications 

In OST, graft rethrombosis, whether early or late, associated with bypass construction, leads to significant morbidity, an increased risk of limb loss, and mortality. The causes of graft rethrombosis are varied and include not only the patient's known risk factors and comorbidities but also technical errors during the reconstruction process. Technical inaccuracies in bypass construction account for early rethrombosis in 20% of cases. The factors determining the success of arterial reconstruction include the quality of inflow and outflow, the size and material of the prosthesis used, surgical technique, and the patient's coagulation profile. A mismatch among these factors jeopardizes the patency of the reconstruction [30].

Based on the timing of their occurrence, rethromboses are classified early (up to 30 days post-bypass construction), most commonly due to technical errors, and late (after 30 days). The usual approach to early rethromboses is open surgical thrombectomy of the bypass, which is technically straightforward, quicker, and less expensive compared to the alternative approach of starting fibrinolytic therapy. The latter requires more time, carries a higher risk of bleeding, and is overall more costly. Sometimes, identifying the technical error can be challenging, and concurrent angiographic examination might be appropriate. In this analysis, approximately 1/5 (20.4%) of the recorded rethromboses are early, equating to nine cases: six cases (13.6%) in surgical treatment, one case (2.3%) in endovascular treatment, and two cases (4.6%) in hybrid treatment. These data suggest a comparability of the three revascularization methods while highlighting the importance of technical execution in direct manual manipulation of the arteries. The frequency of rethromboses (both early and late combined) over a five-year follow-up period is the highest in OST (6.2% of all cases examined), followed by hybrid treatment (1.0%), and lowest in endovascular treatment (0.3%).

Late rethromboses, emerging after 30 days from bypass surgery, stem from different causes. Thrombolytic therapy becomes a key option for these late cases, offering an effective revascularization path. However, open surgery for these rethromboses faces significant challenges due to adhesions and altered anatomy, often requiring complex reoperations. To maintain long-term patency post-thrombectomy, endovascular interventions are frequently employed to address structural graft changes, neointimal hyperplasia, and adjacent atherosclerotic progression. Treatment options include angioplasty, stenting, and patch angioplasty, with late rethrombosis after the initial month observed in 35 cases (79.6%) across all treatment groups in our study, highlighting the necessity of personalized therapeutic strategies based on the timing and cause of rethrombosis.

Re-thromboses are a critical complication across all three surgical strategies, leading to high healthcare and social costs, difficulties in subsequent treatment, and significantly increasing the risk of limb loss and cardiovascular complications. Clinically, rethrombosis becomes significant when it causes definitive necrobiotic changes and limb loss. This study collected data only on major amputations (above the ankle level). Out of 44 re-thrombosis cases, limb amputation occurred in 10 cases, accounting for 22.7% of the rethrombosed reconstructions. By treatment method, the distribution is as follows: one case (2.3%) in the endovascular group, two cases (4.5%) in the HST group, and seven cases (15.9%) in the traditional OST group. Further data analysis revealed that, in two cases leading to limb loss, rethrombosis was associated with local infectious complications necessitating the explantation of the synthetic material used. In three cases, the rethrombosis of the ilio-femoral reconstruction was mediated by insufficient outflow due to femoropopliteal involvement by PAD. Regarding the timing of rethrombosis post-reconstruction, only two cases of limb loss were due to early obstruction, occurring within 30 days (one treated endovascularly and one with constructed ilio-femoral bypass). In the hybrid group, no patient experienced amputation due to early re-thrombosis. The low percentage of early rethromboses indicates comparable technical success among the described treatment methodologies for TASC II C and D lesions in PAD, as well as similar limb salvage rates.

The higher number of rethromboses and amputations over time in surgical and hybrid treatments likely results from the greater use of synthetic material (in surgical treatments), exposure to highly thrombogenic surfaces (after desobliteration in hybrid treatments), and compromise of collaterals (in both methods), unlike in endovascular treatment where these factors are much less prominent. PAD inevitably affects the underlying femoropopliteal segment, playing a crucial role in this complication combination. From the current analysis, the main risk factors for amputation are identified as male gender and advanced age, smoking, multi-focal atherosclerosis (MFA), and stage III and IV disease according to the Fontaine classification. One of the largest population studies investigating major amputation across the three PAD treatment strategies, covering 2325 patients over a 20-year period (1990-2009), was published in 2022. The authors note an increasing frequency of endovascular/hybrid revascularizations and a decreasing frequency of open surgical interventions for PAD, associated with a reduced frequency of major amputations in this population. In the cited study, 82% of 689 open reconstructions involve bypass, most often suprainguinal (39% are infragenicular reconstructions). Stenting accounts for nearly half of the 611 endovascular procedures, predominantly suprainguinal (81%), with less than 1% involving infragenicular vessels. Balloon angioplasty (without stenting) represents 42% of the endovascular patients studied and is most often infragenicular. Hybrid procedures in this analysis are represented by 74 cases (N=74), with hybrid intervention most commonly infragenicular yet supragenicular. Additionally, 82% of these reconstructions aim at multi-stage disease treatment (e.g., femoral endarterectomy plus iliac stenting). When all indications are considered together, more limbs are amputated following endovascular and hybrid treatment than in the group undergoing primary open treatment [31].

When assessing limb loss, the infrainguinal progression of PAD must be considered. In the current analysis, there is only indirect data for femoropopliteal involvement by PAD, based on the 87 infrainguinal reconstructions undertaken after the index iliac reconstruction, which accounted for 15% of all cases studied. This highlights the importance of recognizing PAD's broader impact, particularly its extension beyond the iliac region, which significantly influences treatment strategies and outcomes.

In OST and HST groups, restenoses are another local complication encountered. They are caused by the development of neointimal hyperplasia at the suture line of the anastomosis or patch or by the progression of atherosclerosis in areas adjacent to the suture line [32].

Restenosis is typically identified through the recurrence of claudication symptoms, a decrease in the ABI, or during ultrasound and angiographic diagnostics for another reason. Upon detecting restenosis, the treatment approach considers the type of graft, the length of the newly developed restenotic lesion, its timing of occurrence (early <3-6 months; late >3-6 months), and the patient's comorbidities. Isolated graft restenoses at the site of the proximal anastomosis, developing aix months post-construction, show very good outcomes following balloon angioplasty with or without subsequent stenting. For restenoses at the distal anastomosis in the femoral area, open treatment is preferred - removing the affected segment and interposing a new synthetic graft or patch angioplasty or, in some cases, extending the bypass to a more distal patent segment.

Restenosis is a primary cause of late rethrombosis. While in open surgery with bypass construction restenoses form at the anastomosis sites, in endovascular treatment, they occur around the implanted stents or in adjacent arterial segments due to the progression of systemic disease. In the conducted analysis, the highest percentage of restenoses was expectedly identified in endovascular (14 cases, 32.5% of all identified stenoses) and hybrid treatments (19 cases, 44.18%), with the lowest in surgical treatment (four cases, constituting 13.9% of reported stenotic complications). Likely, their frequency is much higher but is masked by thrombosis due to the often observed neglect of patient complaints in clinical practice and delays in the diagnostic process.

In a literature review published by Kavanagh et al., the cumulative frequency of complications across all studies reviewed is 12.6% [17]. Complications were categorized into those related to the surgical wound (hematoma, infection, bleeding), systemic complications (pneumonia, need for blood transfusion, myocardial infarction), early limb loss, or early occlusion of the reconstruction. Early limb loss was defined as unexpected major amputation within 30 days of the reconstruction. Early occlusions were treated with thrombectomy or conversion to ilio-femoral bypass. The cumulative 30-day mortality reported in the literature was 0.4% [9,16]. Based on the mentioned studies, the calculated frequency of amputations was 1.7% [31]. In the current study, the frequency of complications following HST was 12.56%, with complications categorized into an infection of the surgical wound, rethrombosis, bleeding, complications related to the puncture site, amputation, and death. The frequency of amputations was 1% for HST, with no deaths observed in this treatment method within the representative sample. This demonstrates comparable outcomes for this methodology to those reported globally. Early rethrombosis in endovascular and surgical treatment (<30 days) is most commonly due to technical errors (unverified dissections, residual stenosis) or inaccuracies in the selection of the revascularization method. Another cause is acute thrombosis (due to inadequate anticoagulation) or unrecognized distal embolization. Nonetheless, these complications are rare, with various authors reporting technical success rates of 80-90% [33]. Late rethromboses (>30 days) are caused by new or worsening stenoses, the development of neointimal hyperplasia, and the progression of the atherosclerotic process [34]. 

Unlike surgical treatment, infection is extremely rare in endovascular procedures: frequency <0.4% for stent grafts and <0.1% for stents. The risk factors and treatments are identical to those in surgical treatment. HST combines the best aspects of surgical and endovascular treatment but also carries a combination of complications from both approaches.

Study Limitations

Our study was non-randomized and retrospective, with some patients treated over 15 years ago, resulting in incomplete data. Patients with stenosis were excluded, and follow-up was not comprehensive. Operator preferences and skills influenced treatment choices, introducing bias. The learning curve of surgeons also impacted results. The post-procedural ABI is an objective measure of lower limb perfusion, but it does not assess specific arterial segments or adequately evaluate iliac reconstructions. Systematic ABI assessments were not conducted, limiting conclusions. Data on the infrainguinal spread of PAD were not collected, and 87 subsequent revascularizations did not reflect infrainguinal PAD involvement. Quality of life surveys, though relevant, are rarely used in Bulgaria and were not included, as they represent subjective criteria unsuitable for this retrospective analysis. All patients included in the presented analysis had anatomical types of lesions TASC II C and D, so this parameter was not analyzed due to the assumption that all have practically similar/comparable lesions. The preoperative ABI was not included in the study for two reasons. Firstly, because postoperative monitoring is not part of the protocol for patient follow-up visits, depriving us of the opportunity to compare this important indicator. Secondly, the ABI is an indicator of the overall perfusion of the lower limb, and in cases of multi-level involvement, revascularization of the iliac segment may not improve the ABI due to the presence of simultaneous distal damage in the vascular bed. Only patients with symptomatic PAD stages 2-4 according to the Fontaine classification were subjected to revascularization.

## Conclusions

In the context of an aging population and the increasing severity of risk factors, PAD has become an increasingly significant social issue, with substantial morbidity, disability, and considerable healthcare and economic costs. Understanding the differences in functional outcomes of revascularization adds meaning to the choice of therapeutic strategy and is crucial for the dialogue between doctor and patient, based on the benefits, risks, and costs of the decision.

The development of vascular surgery, focusing on endovascular techniques, reflects current trends in PAD treatment. Despite efforts to promote the primary endovascular strategy, challenges arise when endovascular intervention fails to achieve desired outcomes. In such instances, open surgery, despite its "partial abandonment," continues to be relevant, especially for patients requiring a comprehensive and individualized approach. Hybrid techniques offer a promising solution, combining the advantages of endovascular and open surgery. This method may well represent the future of vascular surgery, providing a balance between minimally invasive methods and traditional surgical intervention, enriching the options for successful treatment. All three revascularization strategies - endovascular, surgical, and hybrid - should be considered complementary elements in the vascular surgeon's toolkit. The goal is to provide optimal and personalized treatment for patients with chronic lower limb ischemia.
